# Dynamic Hyperglycemic Patterns Predict Adverse Outcomes in Patients with Acute Ischemic Stroke Undergoing Mechanical Thrombectomy

**DOI:** 10.3390/jcm9061932

**Published:** 2020-06-20

**Authors:** Giovanni Merlino, Carmelo Smeralda, Massimo Sponza, Gian Luigi Gigli, Simone Lorenzut, Alessandro Marini, Andrea Surcinelli, Sara Pez, Alessandro Vit, Vladimir Gavrilovic, Mariarosaria Valente

**Affiliations:** 1 Stroke Unit, Department of Neuroscience, Udine University Hospital, 33100 Udine, Italy; simone.lorenzut@asufc.sanita.fvg.it; 2 Clinical Neurology, Udine University Hospital, 33100 Udine, Italy; carmelosmeralda@gmail.com (C.S.); gigli@uniud.it (G.L.G.); alemarini00@gmail.com (A.M.); andsurcinelli@gmail.com (A.S.); sarapez91@gmail.com (S.P.); mariarosaria.valente@uniud.it (M.V.); 3 DAME, University of Udine, 33100 Udine, Italy; 4 Division of Vascular and Interventional Radiology, Udine University Hospital, 33100 Udine, Italy; massimo.sponza@asufc.sanita.fvg.it (M.S.); alessandro.vit@asufc.sanita.fvg.it (A.V.); vladimir.gavrilovic@asufc.sanita.fvg.it (V.G.); 5 DMIF, University of Udine, 33100 Udine, Italy

**Keywords:** hyperglycemia, acute ischemic stroke, large vessel occlusion, mechanical thrombectomy

## Abstract

Background: Admission hyperglycemia impairs outcome in acute ischemic stroke (AIS) patients undergoing mechanical thrombectomy (MT). Since hyperglycemia in AIS represents a dynamic condition, we tested whether the dynamic patterns of hyperglycemia, defined as blood glucose levels > 140 mg/dl, affect outcomes in these patients. Methods: We retrospectively analyzed data of 200 consecutive patients with prospective follow-up. Based on blood glucose level, patients were distinguished into 4 groups: (1) persistent normoglycemia; (2) hyperglycemia at baseline only; (3) hyperglycemia at 24-h only; and (4) persistent (at baseline plus at 24-h following MT) hyperglycemia. Results: AIS patients with persistent hyperglycemia have a significantly increased risk of poor functional outcome (OR 6.89, 95% CI 1.98–23.94, *p* = 0.002, for three-month poor outcome; OR 11.15, 95% CI 2.99–41.52, *p* = 0.001, for no major neurological improvement), mortality (OR 5.37, 95% CI 1.61–17.96, *p* = 0.006, for in-hospital mortality; OR 4.43, 95% CI 1.40–13.97, *p* = 0.01, for three-month mortality), and hemorrhagic transformation (OR 6.89, 95% CI 2.35–20.21, *p* = 0.001, for intracranial hemorrhage; OR 5.42, 95% CI 1.54–19.15, *p* = 0.009, for symptomatic intracranial hemorrhage) after endovascular treatment. These detrimental effects were partially confirmed after also excluding diabetic patients. The AUC-ROC showed a very good performance for predicting three-month poor outcome (0.76) in-hospital mortality (0.79) and three-month mortality (0.79). Conclusions: Our study suggests that it is useful to perform the prolonged monitoring of glucose levels lasting 24-h after MT.

## 1. Background

Mechanical thrombectomy (MT) is the first-line treatment for acute ischemic stroke (AIS) due to large vessel occlusion (LVO) [1]. Several randomized, controlled trials reported that MT improves the outcome, in comparison with the best medical therapy [2,3,4,5,6]. In these studies, the prevalence of good outcome at three months was as high as 71% [2,3,4,5,6]. However, data coming from observational registries reported a significantly lower rate of patients with functional independence; this ranged between 34% and 39% [7,8,9]. In addition to blood pressure control and time to reperfusion, the glycemic status represents one of the most important modifiable predictors of adverse outcomes in patients undergoing MT [10,11,12].

Hyperglycemia impairs outcome in patients with AIS [13,14,15]. Among the several mechanisms implicated in this unfavorable association, it is important to remember that increased glucose levels in stroke patients alter the blood barrier permeability [16], exacerbate the thromboinflammatory cascade [17], induce acidosis [18], and increase oxidative stress response [19]. In AIS patients treated with alteplase, admission hyperglycemia has been associated with the increased risk of death, symptomatic intracranial hemorrhage (SICH), and poor functional status [20].

In recent years, the interest of the international scientific community shifted towards the role of altered glycemic status in affecting the outcome of AIS patients undergoing MT for LVO. Several studies observed that admission hyperglycemia reduced the likelihood of a good outcome in AIS patients treated with MT [21,22,23,24]. However, hyperglycemia may also occur during the post-operative period, both in diabetic and non-diabetic patients. A stress-response, characterized by excessive gluconeogenesis, glycogenolysis and insulin resistance with consequent hyperglycemia, is common after large strokes [25]. Recently, Li et al. investigated the role of post-operative hyperglycemia as a potential predictor of SICH in AIS patients treated with MT. The authors observed that, differently from glucose levels at admission, post-operative hyperglycemia increased the risk of the occurrence of SICH and parenchymal hematoma (PH) [26].

Since hyperglycemia in AIS represents a dynamic condition, we hypothesize that one isolated glucose test measure, performed at admission or within 24-h after MT, might be insufficient to understand the effects of the metabolic process on the ischemic brain. To date, only a few studies investigated the contribution of the dynamic patterns of hyperglycemia to stroke outcome [27,28,29]. These trials included only AIS patients treated and not treated with alteplase, whereas similar investigations in patients undergoing MT are lacking. We decided to perform this study with the aim of evaluating the impact of the dynamic patterns of hyperglycemia on stroke outcome, in AIS patients with LVO who were treated with MT.

## 2. Methods

### 2.1. Patients

This study is a retrospective analysis of consecutive patients with prospective follow-up admitted to the Udine University Hospital with AIS due to LVO, that were treated with MT from January 2015 to December 2019. Eligibility criteria for MT were the following: (1) presence of LVO in the anterior or posterior circulation, as revealed by CT angiography; (2) symptoms onset within 6 h for LVO in the anterior circulation and within 8 h for LVO in the posterior one; and (3) Alberta Stroke Program Early CT Score (ASPECTS) > 6 on direct CT scan. In our center, the following exclusion criteria for MT are in use: (1) life expectancy less than 12 months; (2) severe internal medicine diseases with signs of organ failure; and (3) platelet count less than 55,000 mmc. All patients treated with MT in our center were included in this study. No specific exclusion criteria were adopted. Patients showing symptoms onset within 4.5 h received alteplase in accordance with the international guidelines [1].

The study was approved by our local Ethics Committee (Ref. No. CEUR-2020-Os-173). Informed consent was obtained from the participants in this study, or their representatives.

### 2.2. Data Collection

The following variables were collected: age, sex, vascular risk factors, laboratory findings, including glycated hemoglobin (HbA1c), admission systolic blood pressure, and pharmacological treatment.

### 2.3. Vascular Risk Factors

Based on previous studies [30,31,32], we adopted the following definitions of vascular risk factors: (1) previous transient ischemic attack/stroke was defined if the patient had a history of ischemic (transient attack or stroke) or hemorrhagic cerebrovascular disease; (2) the presence of cardiovascular disease was based on the history of previous ischemic heart disease and/or revascularization treatment using percutaneous coronary intervention/coronary artery bypass grafting; (3) atrial fibrillation was defined if the patient had past medical history of atrial fibrillation that had been confirmed in medical records; (4) high blood pressure was defined as the history of hypertension and/or use of antihypertensive medication; (5) a history of diabetes mellitus that had been confirmed in medical records and/or use of insulin/oral hypoglycemic agents were considered for defining diabetes; (6) a presence of hypercholesterolemia was based on the use of lipid-lowering medications; (7) information on active tobacco use was used for defining patient as a current smoker.

### 2.4. Measurement of Blood Glucose

Blood glucose was measured at admission (baseline glucose level), before initiating any specific stroke treatment, and within 24 h after MT (post-operative glucose level). Based on previous studies on this topic, hyperglycemia was defined as a glucose level > 140 mg/dL [21,22,23]. Based on blood glucose level, patients were distinguished into 4 groups: (1) persistent normoglycemia, i.e., normoglycemia at baseline plus at 24-h; (2) hyperglycemia at baseline only; (3) hyperglycemia at 24-h only; and (4) persistent hyperglycemia, i.e., hyperglycemia at baseline plus at 24-h.

### 2.5. Clinical Assessment

#### 2.5.1. Trial of ORG 10,172 in Acute Stroke Treatment classification

The trial of ORG 10,172 in acute stroke treatment (TOAST) classification was used to determine AIS subtypes based on their etiology. In particular, cerebrovascular events were distinguished as due to large artery atherosclerosis, cardioembolism, small-vessel occlusion, other determined etiology, and undetermined etiology [33].

#### 2.5.2. National Institute of Health Stroke Scale Score

Stroke severity was determined with the National Institute of Health Stroke Scale (NIHSS) score, at admission and at discharge [34]. In accordance with many previous studies, we defined patients with major neurological improvement as those who had an improvement of ≥8 points on the NIHSS from baseline or a NIHSS score of 0 at discharge [35,36,37].

#### 2.5.3. Modified Rankin Scale

Functional outcome was assessed by means of the modified Rankin scale (mRS) at admission based on pre-stroke disability and 3 months after stroke [38]. The mRS score after discharge was recorded at the patients’ routine clinical visit or through telephone interviews with the patients or their immediate caregivers. The mRS score was dichotomized into: favorable outcome (0–2) and poor outcome (3–6).

#### 2.5.4. Hemorrhagic Transformation

The presence of intracranial hemorrhage (ICH) was defined as any PH based on the European Cooperative Acute Stroke Study (ECASS) morphologic definitions (ECASS PH-1 or PH-2) [39], whereas the presence of SICH was based on the ECASS-III protocol [40].

### 2.6. Thrombectomy Procedure

We collected the following information: site of the cerebral artery occlusion, distinguished in middle cerebral artery (MCA), tandem (ICA + MCA), and vertebrobasilar; type of device used for MT procedure, classified as thromboaspiration, stent retriever, thromboaspiration plus stent retriever, and permanent stenting; presence or absence of secondary embolization; time from symptom onset to MT; procedure duration; recanalization rate, assessed at the end of MT, using the thrombolysis in cerebral infarction (TICI) classification and defined as successful recanalization when a TICI 2b-3 was achieved.

### 2.7. Outcome Measures

The following endpoints were analyzed: 3-month poor outcome, no major neurological improvement at discharge, in-hospital mortality, 3-month mortality, presence of ICH, and presence of SICH. All the outcome measures were collected as part of our routine clinical practice in patients affected by cerebrovascular events.

### 2.8. Statistical Analysis

Data are displayed in tables as median and interquartile range (IQR).

Differences between the 4 groups were assessed by means of the Chi square test or the Fisher’s exact test, when appropriate, for categorial variables. One-way analysis of variance for normally distributed continuous variables, and the Kruskal–Wallis test for non-normally distributed continuous variables and for ordinal variables were used. Post-hoc analysis was performed by means of the Bonferroni test. The Kolmogorov–Smirnov test with Lilliefors significant correction was used to assess the normal distribution of data.

Multiple logistic regression analysis was performed to test the impact of hyperglycemia risk groups, with reference to the normoglycemia group. The potential confounding variables included in the model were: age, HbA1c values, use of antidiabetic drugs, intravenous thrombolysis before MT, baseline NIHSS score, pre-stroke mRS, time from symptom onset to MT, and successful recanalization. Systolic blood pressure > 180 mmHg was added to other confounders in the analysis, that evaluated the association between hyperglycemic patterns and hemorrhagic transformation (i.e., ICH, SICH) [1]. Multivariate analysis was performed for all the sample population and, later, only for patients without diabetes (subjects with a history of diabetes and/or with HbA1c values ≥ 6.5% were excluded from this analysis).

The utility of the hyperglycemic patterns in estimating unfavorable outcomes was tested with area under the receiver operating characteristic curve (AUC-ROC).

All probability values are two-tailed. A *p* value < 0.05 was considered statistically significant. Statistical analysis was carried out using the SPSS Statistics, Version 22.0 (Chicago, IL, USA).

## 3. Results

### 3.1. Baseline Characteristics

Among the 200 patients recruited during the study period, 116 (58%) had persistent normoglycemia, whereas 36 (18%) had elevated glucose only at baseline, 17 (8.5%) only at 24-h, and 31 (15.5%) persistent hyperglycemia. Oral antidiabetic agents were taken by 21 patients (10.5%), whereas only two patients were treated with insulin.

The general characteristics of the enrolled subjects are presented in Table 1. We did not observe any difference regarding age and sex between the 4 groups. The prevalence of diabetes mellitus and hypercholesterolemia was significantly higher in patients with persistent hyperglycemia than in the other three groups (*p* = 0.001). In addition, more than 80% of patients with persistent hyperglycemia was affected by hypertension. Compared to patients with persistent normoglycemia and baseline hyperglycemia, HbA1c values were significantly higher among subjects with persistent hyperglycemia (*p* = 0.001). Furthermore, these patients took slightly more antiplatelets. The use of alteplase before MT was similar among the groups. While admission NIHSS score was not different among the 4 groups, stroke severity at discharge was largely increased in patients with baseline and persistent hyperglycemia (*p* = 0.04).

Table 2 summarizes information on MT in the four groups. No significant difference was observed among the groups. As expected, MCA was the most common site of LVO in the four groups. The combined technique using thromboaspiration plus stent retriever was adopted in a large part of our sample (43.5%), whereas thrombectomy only with stent retriever was performed in only 6% of the patients. The median time between symptoms onset and MT was almost 220 min, while the procedure length was 70 min. The prevalence of successful recanalization was as high as 85%.

### 3.2. Association of Hyperglycemic Patterns with Clinical Outcomes in Univariate Analysis

**Figure 1:**
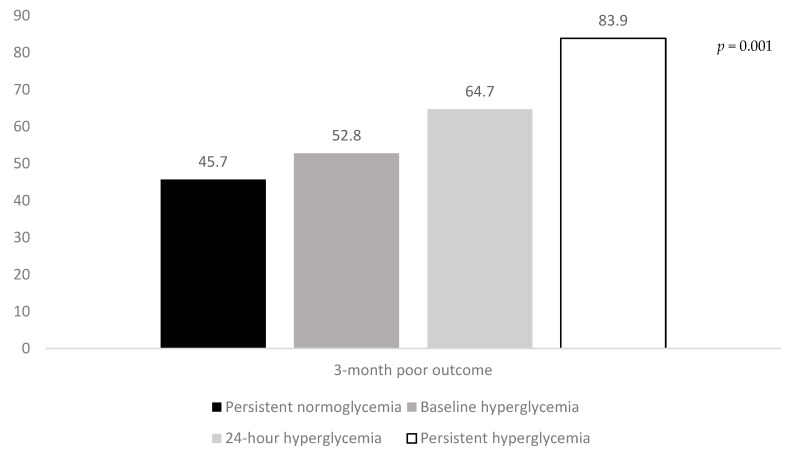
Rates of three-month poor outcome according to the hyperglycemic patterns.

**Figure 2:**
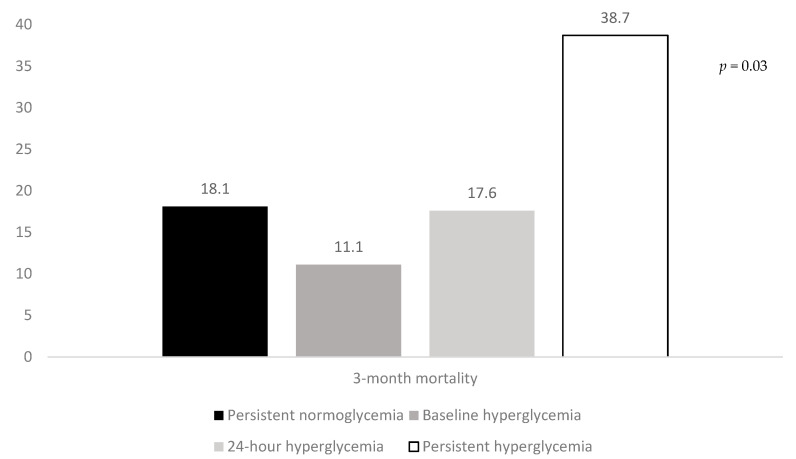
Rates of three-month mortality according to the hyperglycemic patterns.

**Figure 3:**
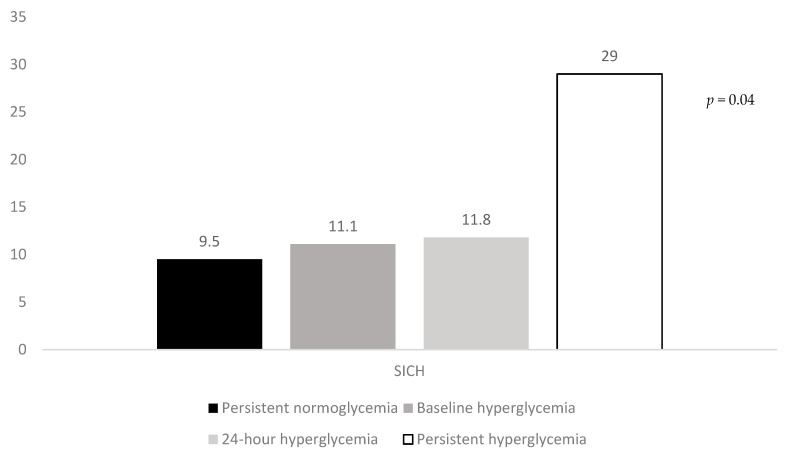
Rates of SICH according to the hyperglycemic patterns. SICH: symptomatic intracranial hemorrhage.

The rates of three-month poor outcome, three-month mortality, and SICH according to the hyperglycemic patterns are reported in Figure 1, Figure 2 and Figure 3. The rates of three-month poor outcome, three-month mortality, and SICH prevalence of no major neurological improvement (24% for persistent normoglycemia, 55.9% for baseline hyperglycemia, 57.1% for 24-h hyperglycemia, and 66.7% for persistent hyperglycemia, *p* = 0.001), in-hospital mortality (10.3% for persistent normoglycemia, 5.6% for baseline hyperglycemia, 17.6% for 24-h hyperglycemia, and 32.3% for persistent hyperglycemia, *p* = 0.006), and ICH (19% for persistent normoglycemia, 22.2% for baseline hyperglycemia, 11.8% for 24-h hyperglycemia, and 54.8% for persistent hyperglycemia, *p* = 0.001) were statistically different among the four groups.

### 3.3. Association of Hyperglycemic Patterns with Clinical Outcomes in Multivariate Analysis

As reported in Table 3, all the outcomes were significantly associated with the presence of persistent hyperglycemia, even after controlling for confounders. Independent predictors, other than persistent hyperglycemia, were the following: (1) NIHSS score at admission (OR 1.11, 95% CI 1.04–1.18, *p* = 0.002) for three-month poor outcome; (2) baseline hyperglycemia (OR 3.37, 95% CI 1.39–8.19, *p* = 0.007), time from symptoms onset to MT (OR 1.01, 95% CI 1.00–1.01, *p* = 0.02), and successful recanalization (OR 0.22, 95% CI 0.07–0.66, *p* = 0.007) for no major neurological improvement; (3) age (OR 1.06, 95% CI 1.01–1.13, *p* = 0.05), NIHSS score at admission (OR 1.13, 95% CI 1.01–1.27, *p* = 0.04), and successful recanalization (OR 0.31, 95% CI 0.09–0.98, *p* = 0.04) for in-hospital mortality; (4) age (OR 1.08, 95% CI 1.02–1.13, *p* = 0.008), and pre-stroke mRS (OR 1.52, 95% CI 1.08–2.14, *p* = 0.02) for three-month mortality; systolic blood pressure > 180 mmHg for both (5) ICH (OR 3.33, 95% CI 1.43.31–7.79, *p* = 0.005), and (6) SICH (OR 9.69, 95% CI 3.55–26.47, *p* = 0.001).

As summarized in Table 4, persistent hyperglycemia was independently associated with the outcome measures, with the exception of in-hospital mortality and three-month mortality, in non-diabetic patients. The independent predictors, other than persistent hyperglycemia, were the following: (1) age (OR 1.04, 95% CI 1.00–1.08, *p* = 0.03), and NIHSS score at admission (OR 1.12, 95% CI 1.04–1.20, *p* = 0.002) for three-month poor outcome; (2) baseline hyperglycemia (OR 4.43, 95% CI 1.57–12.53, *p* = 0.005), 24-h hyperglycemia (OR 4.29, 95% CI 1.01–12.53, *p* = 0.05), time from symptoms onset to MT (OR 1.01, 95% CI 1.00–1.01, *p* = 0.04), and successful recanalization (OR 0.23, 95% CI 0.06–0.84, *p* = 0.03) for no major neurological improvement; (3) NIHSS score at admission (OR 1.19, 95% CI 1.03–1.37, *p* = 0.01), and successful recanalization (OR 0.19, 95% CI 0.04–0.67, *p* = 0.01) for in-hospital mortality; (4) age (OR 1.08, 95% CI 1.02–1.15, *p* = 0.009), and pre-stroke mRS (OR 1.49, 95% CI 1.01–2.20, *p* = 0.04) for three-month mortality; and systolic blood pressure > 180 mmHg for both (5) ICH (OR 3.76, 95% CI 1.42–9.97, *p* = 0.008), and (6) SICH (OR 6.91, 95% CI 2.28–20.95, *p* = 0.001).

### 3.4. Predictive Value of Hyperglycemic Patterns

In order to evaluate the diagnostic performance of the hyperglycemic patterns, as judged with AUC-ROC, we added them to the basic model that included the other independent predictors of each endpoint. After adding the hyperglycemic patterns to the basic model, the AUC-ROC value increased for predicting a three-month poor outcome from 0.70 (95% CI 0.63–0.77) to 0.76 (95% CI 0.69–0.82), for no major neurological improvement from 0.63 (95% CI 0.55–0.72) to 0.75 (95% CI 0.67–0.82), for in-hospital mortality from 0.69 (95% CI 0.59–0.78) to 0.79 (95% CI 0.71–0.87), for three-month mortality from 0.72 (95% CI 0.63–0.81) to 0.79 (95% CI 0.71–0.86), for ICH from 0.62 (95% CI 0.52–0.71) to 0.70 (95% CI 0.61–0.79), and for SICH from 0.71 (95% CI 0.59–0.83) to 0.77 (95% CI 0.66–0.88).

## 4. Discussions

In order to evaluate the impact of the dynamic patterns of hyperglycemia on stroke outcome in AIS patients with LVO treated with MT, we retrospectively analyzed the results observed in a series of consecutive patients with prospective follow-up. We demonstrated that AIS patients affected by LVO with persistent hyperglycemia, defined as blood glucose levels > 140 mg/dL at baseline plus at 24-h following MT, have a significantly increased risk of poor functional outcome, mortality, and hemorrhagic transformation after endovascular treatment. These detrimental effects were partially confirmed after also excluding diabetic patients.

Previous studies performed in AIS patients treated with MT used one isolated glucose test measurement at baseline for investigating the role of hyperglycemia as a predictor of unfavorable outcomes [21,22,23,24]. Taken together, results on admission hyperglycemia as a predictor of poor outcome in patients undergoing MT are contradictory, with some articles showing that hyperglycemia at admission was a predictor of a worse outcome [22,24], and others showing no effect of blood glucose levels [21,23]. To date, only one study investigated whether hyperglycemia after MT was associated with a worse outcome. Li et al. analyzed 156 patients and reported that post-operative hyperglycemia (1 mmol/L per increase, i.e., 18 mg/dl per increase) was an independent predictor of SICH (OR 1.20, 95% CI 1.06–1.36, *p* = 0.008) [26].

Differently from previous studies, we sought to investigate the clinical significance of the dynamic patterns of hyperglycemia in patients undergoing MT. Although no other study has been performed in this specific population, a post-hoc analysis of the ECASS-II trial lead the authors to conclude that, in addition to a single glucose measurement, the pattern of glycemic excursions should be considered in the prediction of stroke outcome [27]. A further study by Putaala et al. investigated 851 consecutive patients treated with alteplase. Differently from baseline normoglycemia, persistent and 48-h hyperglycemia predicted an unfavorable outcome, death, and SICH [28]. Similar results were reported by Yoo et al. [29]

Our findings show that the presence of glucose levels > 140 mg/dL at admission, plus at 24-h after MT, has harmful and detrimental effects, also in non-diabetic patients. It is very unlikely that this result may have been influenced by the use of alteplase prior to MT, since the use of alteplase was included in our multivariate analysis as confounder. A large number of patients with persistent hyperglycemia was functionally dependent at 3 months after stroke, their in-hospital and three-month mortality were as high as 32.3% and 38.7%, respectively, and finally, almost 30% of these patients experienced SICH. Apart from the endpoint called “no major neurological improvement at discharge”, patients with isolated admission hyperglycemia did not show any increased risk of adverse outcomes. Similarly, isolated hyperglycemia at 24-h after MT was not associated with a worse functional outcome, as well as a higher likelihood of death and SICH. Persistent hyperglycemia also had detrimental effects in non-diabetic patients. In fact, patients with this abnormal glycemic pattern had a higher risk of functional dependence and hemorrhagic transformation than those with persistent normoglycemia. Differently, the presence of persistent hyperglycemia was not associated with in-hospital and three-month mortality among non-diabetics undergoing MT for AIS.

In our sample, persistent hyperglycemia might impair outcomes as a marker of diabetes mellitus. In fact, patients with persistent hyperglycemia were more frequently affected by the chronic metabolic disease and they showed values of HbA1c significantly higher than subjects with different glycemic profiles. Epidemiologic studies have shown that diabetes is a well-established independent, but modifiable risk factor for stroke; both ischemic and hemorrhagic stroke [41]. There are several possible mechanisms wherein diabetes leads to stroke, among them diabetic vasculopathy could be considered. This pathophysiological hypothesis would be in agreement with the observed association between persistent hyperglycemia and hypercholesterolemia, a known risk factor for atherosclerosis. Furthermore, AIS patients with persistent hyperglycemia took slightly more antiplatelets. Although several clues point to diabetes as the link between persistent hyperglycemia and adverse outcomes in patients undergoing MT, nevertheless, the metabolic disorder alone cannot explain the worse prognosis of hyperglycemic patients. In fact, HbA1c values did not represent an independent predictor of adverse outcomes in the multivariate analysis of our study.

Hyperglycemia occurs after an acute stress, such as stroke and myocardial infarction, by the activation of the hypothalamic-pituitary-adrenal axis [42,43]. Both human and animal studies showed that mortality rate, due to post-stress hyperglycemia, is high after both stroke and myocardial infarction [44]. Previous studies reported that post-stress hyperglycemia was strictly associated with stroke severity. Blood glucose increase was related to the severity of stroke in the study of Christensen et al. [45] Our results, regarding NIHSS score at discharge, are perfectly in line with these previous data. Moreover, we observed that persistent hyperglycemia was able to impair functional outcome and hemorrhagic transformation in non-diabetics. Thus, we postulate that post-stress hyperglycemia not only may occur, but also may cause severe consequences in AIS patients treated with MT.

The AUC-ROC analysis strongly supports the use of hyperglycemic patterns in order to predict outcomes in AIS patients with LVO undergoing MT.

Although our study would encourage to apply rigorous glycemic control for at least 24-h following MT to achieve better outcomes, to date, no evidence supports the concept that ensuring strict post-stroke normoglycemia improves outcome. A possible reason for this might have been the severe hypoglycemia occurred in the intravenous insulin treatment group [46]. In order to overcome the failing of insulin, a phase 2 trial (the TEXAIS trial) on exenatide is now enrolling AIS patients in Australia, New Zealand, and Finland [47].

There are several limitations in our study. This is an observational study performed in a single center with a limited simple size. Although the study was retrospective, we prospectively collected data in consecutive patients. Information on the use of lowering glucose drugs during hospitalization is lacking. In addition, even after controlling for known confounders, residual confounding from unobserved factors might have affected our results. Information on last food intake before admission blood sample was not collected and the lack of a common criterion might have changed values, depending on the length of previous fasting. Unfortunately, no information other than NIHSS score were collected on clinical conditions at discharge, thus limiting the possibility of excluding their influence on measures of follow up at three months. Finally, the sample size was too small for performing further statistical analysis, e.g., propensity matching analysis, able to confirm our preliminary results.

In conclusion, we demonstrated the utility of performing prolonged monitoring of glucose levels lasting 24-h after MT, instead of isolated blood glucose measurements. Further studies are needed to confirm these results in larger samples.

## Figures and Tables

**Table 1:** General characteristics of the subjects according to the hyperglycemic patterns.

	Persistent Normoglycemia	Baseline Hyperglycemia	24-h Hyperglycemia	Persistent Hyperglycemia	*p*
	(*n* = 116)	(*n* = 36)	(*n* = 17)	(*n* = 31)	
**Demographic data**					
Age, years	73 (67–80)	75 (68.2–82)	75 (66.5–78.5)	72 (69–79)	0.7
Males, *n* (%)	61 (52.6)	14 (38.9)	7 (41.2)	19 (61.3)	0.2
**Vascular risk factors**					
Previous transient ischemic attack/stroke, *n* (%)	11 (9.5)	2 (5.6)	3 (17.6)	3 (9.7)	0.6
Cardiovascular disease, *n* (%)	22 (19.0)	7 (19.4)	3 (17.6)	5 (16.1)	0.9
Atrial fibrillation, *n* (%)	31 (26.7)	13 (36.1)	3 (17.6)	6 (19.4)	0.4
Hypertension, *n* (%)	83 (72.2)	26 (72.2)	13 (76.5)	26 (83.9)	0.6
Diabetes mellitus, *n* (%)	7 (6.0)	6 (16.7)	3 (17.6)	13 (41.9)	0.001
Hypercholesterolemia, *n* (%)	25 (21.6)	9 (25.0)	1 (5.9)	13 (41.9)	0.03
Current smoking, *n* (%)	21 (18.1)	7 (19.4)	5 (29.4)	9 (29.0)	0.5
**Laboratory findings**					
HbA1c values, %	5.7 (5.4–6.0)	6.0 (5.7–6.4)	6.0 (5.9–6.4)	6.4 (5.8–7.1)	0.001
Total cholesterol, mg/dL	164 (145–195.5)	171 (145–194)	166 (122.2–206.7)	154 (135.2–170)	0.5
HDL cholesterol, mg/dL	51 (41–62)	50 (41–63.2)	47.5 (30.75–62.2)	46 (39–59)	0.7
LDL cholesterol, mg/dL	95 (77–121.2)	95.5 (80–121.2)	94.5 (67.2–124.5)	87 (68–101)	0.8
Triglycerides, mg/dL	92 (70–130.5)	85 (63–123)	73.5 (56.7–139)	95 (67.5–143.5)	0.4
**Blood pressure**					
Systolic blood pressure, mmHg	151 (130–170)	155 (143–163)	154 (145–168)	155 (138–180)	0.7
**Antithrombotic treatment at admission**					
Antiplatelets, *n* (%)	28 (24.1)	13 (36.1)	5 (29.4)	14 (45.2)	0.1
Anticoagulants, *n* (%)	16 (13.8)	6 (16.7)	2 (11.8)	3 (9.7)	0.9
**Stroke subtypes based on TOAST classification**					0.6
Large arterial atherosclerosis, *n* (%)	19 (16.4)	6 (16.7)	2 (11.8)	6 (19.4)	
Cardioembolism, *n* (%)	60 (51.7)	16 (44.4)	10 (58.8)	14 (45.2)	
Other determined etiology, *n* (%)	6 (5.2)	0 (0.0)	0 (0.0)	0 (0.0)	
Undetermined etiology, *n* (%)	31 (26.7)	14 (38.9)	5 (29.4)	11 (35.5)	
**Baseline clinical characteristics**					
Alteplase use before MT, *n* (%)	66 (56.9)	22 (61.1)	8 (47.1)	20 (64.5)	0.7
NIHSS score at admission	16.5 (13–20)	19 (15.2–22)	17 (14.5–19.5)	18 (15–22)	0.2
NIHSS score at discharge	3 (1–8.7)	9 (2–16.7)	7.5 (1.7–17.7)	12 (2.5–16.5)	0.04
Pre-stroke mRS 0–2, *n* (%)	103 (88.8)	34 (94.4)	17 (100)	28 (90.3)	0.4

HbA1c: glycated hemoglobin; MT: mechanical thrombectomy; NIHSS: National Institute of Health Stroke Scale; mRS: modified Rankin Scale.

**Table 2:** Information on mechanical thrombectomy, according to the hyperglycemic patterns.

	Persistent Normoglycemia	Baseline Hyperglycemia	24-h Hyperglycemia	Persistent Hyperglycemia	*p*
	(*n* = 116)	(*n* = 36)	(*n* = 17)	(*n* = 31)	
**Site of LVO**					0.5
MCA, *n* (%)	80 (69.0)	22 (61.1)	13 (76.5)	24 (77.4)	
Tandem, *n* (%)	26 (22.4)	7 (19.4)	3 (17.6)	5 (16.1)	
Vertebrobasilar, *n* (%)	10 (8.6)	7 (19.4)	1 (5.9)	2 (6.5)	
**Type of device use for MT**					0.6
Thromboaspiration, *n* (%)	42 (36.2)	11 (30.6)	6 (35.3)	9 (29)	
Stent retriever, *n* (%)	5 (4.3)	2 (5.6)	2 (11.8)	3 (9.7)	
Thromboaspiration *plus* stent retriever, *n* (%)	49 (42.2)	17 (47.2)	9 (52.9)	12 (38.7)	
Permanent stenting, *n* (%)	20 (17.2)	6 (16.7)	0 (0.0)	7 (22.6)	
**Other information on MT**					
Secondary embolization, *n* (%)	6 (5.2)	4 (11.1)	3 (17.6)	3 (9.7)	0.3
Time from symptoms onset to MT, min	210 (170–260)	236 (205–270)	225 (195–310)	210 (155–255)	0.08
Procedure length, min	67.5 (50–98.7)	70 (50–85)	70 (42.5–95)	65 (40–120)	0.9
Successful recanalization rate, *n* (%)	102 (87.9)	29 (80.6)	13 (76.5)	26 (83.9)	0.5

MT: Mechanical thrombectomy; LVO: large vessel occlusion; MCA: middle cerebral artery.

**Table 3:** Logistic regression model: adjusted ORs (95% CIs) of hyperglycemic patterns, in relation to the respective outcomes.

	Persistent Normoglycemia	Baseline Hyperglycemia	24-h Hyperglycemia	Persistent Hyperglycemia
Three-month poor outcome ^†^	1	0.99 (0.41–2.38)	1.75 (0.54–5.67)	6.89 (1.98–23.94) *p* = 0.002
No major neurological improvement at discharge ^†^	1	3.37 (1.39–8.19) *p* = 0.007	3.41 (0.96–12.16)	11.15 (2.99–41.52) *p* = 0.001
In-hospital mortality ^†^	1	0.39 (0.07–2.12)	2.67 (0.58–12.30)	5.37 (1.61–17.96) *p* = 0.006
Three-month mortality ^†^	1	0.50 (0.14–1.82)	1.33 (0.31–5.66)	4.43 (1.40–13.97) *p* = 0.01
Presence of ICH ^‡^	1	1.15 (0.43–3.08)	0.45 (0.08–2.44)	6.89 (2.35–20.21) *p* = 0.001
Presence of SICH ^‡^	1	1.25 (0.33–4.71)	1.31 (0.21–8.31)	5.42 (1.54–19.15) *p* = 0.009

ICH: intracranial hemorrhage; SICH: symptomatic intracranial hemorrhage. ^†^ Adjusted for age, HbA1c values, use of antidiabetic drugs, intravenous thrombolysis, baseline NIHSS score, pre-stroke mRS, time from symptom onset to endovascular treatment, and successful recanalization. ^‡^ Adjusted for age, HbA1c values, use of antidiabetic drugs, intravenous thrombolysis, baseline NIHSS score, pre-stroke mRS, time from symptom onset to endovascular treatment, successful recanalization, and systolic blood pressure > 180 mmHg.

**Table 4:** Logistic regression model in non-diabetic patients: adjusted ORs (95% CIs) of hyperglycemic patterns in relation to the respective outcomes.

	Persistent Normoglycemia	Baseline Hyperglycemia	24-h Hyperglycemia	Persistent Hyperglycemia
three-month poor outcome ^†^	1	1.20 (0.43–3.31)	3.01 (0.75–12.70)	4.91 (1.15–20.94) *p* = 0.03
No major neurological improvement at discharge ^†^	1	4.43 (1.57–12.53) *p* = 0.005	4.29 (1.01–12.53) *p* = 0.05	8.62 (2.01–36.99) *p* = 0.004
In-hospital mortality ^†^	1	0.25 (0.02–2.47)	3.27 (0.57–18.64)	2.80 (0.65–12.08)
three-month mortality ^†^	1	0.49 (0.11–2.11)	1.24 (0.26–5.97)	1.81 (0.44–7.45)
Presence of ICH ^‡^	1	0.93 (0.28–3.03)	0.16 (0.01–1.84)	7.15 (1.98–25.76) *p* = 0.003
Presence of SICH ^‡^	1	1.26 (0.29–5.52)	1.27 (0.18–9.07)	8.26 (1.95–35.01) *p* = 0.004

ICH: intracranial hemorrhage; SICH: symptomatic intracranial hemorrhage. ^†^ Adjusted for age, use of antidiabetic drugs, intravenous thrombolysis, baseline NIHSS score, pre-stroke mRS, time from symptom onset to treatment, and successful recanalization. ^‡^ Adjusted for age, use of antidiabetic drugs, intravenous thrombolysis, baseline NIHSS score, pre-stroke mRS, time from symptom onset to treatment, successful recanalization and systolic blood pressure > 180 mmHg.
